# Progression to lung fibrosis in severe COVID-19 patients: A morphological and transcriptomic study in postmortem samples

**DOI:** 10.3389/fmed.2022.976759

**Published:** 2022-11-03

**Authors:** Belén Pérez-Mies, Tamara Caniego-Casas, Tommaso Bardi, Irene Carretero-Barrio, Amparo Benito, Mónica García-Cosío, Irene González-García, David Pizarro, Marta Rosas, Eva Cristóbal, Yolanda Ruano, María Concepción Garrido, Juan Rigual-Bobillo, Raúl de Pablo, Juan Carlos Galán, David Pestaña, José Palacios

**Affiliations:** ^1^Pathology, Hospital Universitario Ramón y Cajal, Madrid, Spain; ^2^Instituto Ramon y Cajal de Investigación Sanitaria, Madrid, Spain; ^3^Centro de Investigación Biomédica en Red de Cáncer (CIBERONC), Instituto de Salud Carlos III, Madrid, Spain; ^4^Faculty of Medicine, Alcalá University, Alcalá de Henares, Spain; ^5^Department of Anesthesiology and Surgical Critical Care, Hospital Ramón y Cajal, Madrid, Spain; ^6^Department of Pathology, Medical School, Universidad Complutense, Instituto i + 12, Hospital Universitario 12 de Octubre, Madrid, Spain; ^7^Department of Respiratory, Hospital Universitario Ramón y Cajal, Madrid, Spain; ^8^Medical Intensive Care Unit, Hospital Ramón y Cajal, Madrid, Spain; ^9^Clinical Microbiology Unit, Hospital Ramón y Cajal, Madrid, Spain; ^10^Centro de Investigación Biomédica en Red en Epidemiología y Salud Pública (CIBERESP), Madrid, Spain

**Keywords:** COVID-19, fibrosis, transcriptomic, diffuse alveolar damage (DAD), autopsy

## Abstract

The development of lung fibrosis is a major concern in patients recovered from severe COVID-19 pneumonia. This study aimed to document the evolution of diffuse alveolar damage (DAD) to the fibrosing pattern and define the transcriptional programs involved. Morphological, immunohistochemical and transcriptional analysis were performed in lung samples obtained from autopsy of 33 severe COVID-19 patients (median illness duration: 36 days). Normal lung and idiopathic pulmonary fibrosis (IPF) were used for comparison. Twenty-seven patients with DAD and disease evolution of more than 2 weeks had fibrosis. Pathways and genes related with collagen biosynthesis and extracellular matrix (ECM) biosynthesis and degradation, myofibroblastic differentiation and epithelial to mesenchymal transition (EMT) were overexpressed in COVID-19. This pattern had similarities with that observed in IPF. By immunohistochemistry, pathological fibroblasts (pFBs), with CTHRC1 and SPARC expression, increased in areas of proliferative DAD and decreased in areas of mature fibrosis. Immunohistochemical analysis demonstrated constitutive expression of cadherin-11 in normal epithelial cells and a similar pattern of cadherin and catenin expression in epithelial cells from both normal and COVID-19 samples. Transcriptomic analysis revealed downregulation of the Hippo pathway, concordant with the observation of YAP overexpression in hyperplastic alveolar epithelial cells. Progression to fibrosis in severe COVID-19 is associated with overexpression of fibrogenic pathways and increased in CTHRC1- and SPARC-positive pFBs. Whereas the Hippo pathway seemed to be implicated in the response to epithelial cell damage, EMT was not a major process implicated in COVID-19 mediated lung fibrosis.

## Graphical Abstract

Progression to lung fibrosis in COVID-19 patients is related to an overexpression of fibrogenic pathways. 
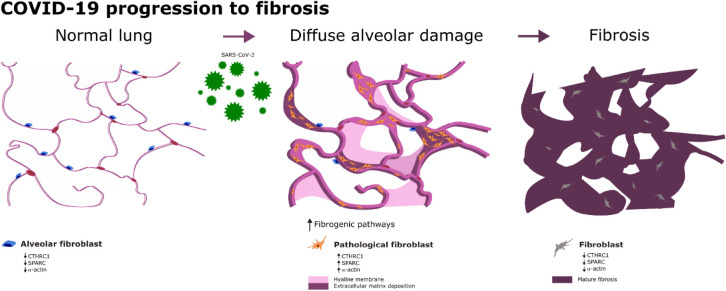

## Introduction

Autopsy studies of COVID-19 patients have demonstrated that, while SARS-CoV-2 can be detected in different organs, the primary finding associated with the cause of death is respiratory failure due to diffuse alveolar damage (DAD) in different stages of evolution. Although most patients show DAD in the exudative or organizing phase, up to 40% of autopsied patients with long term hospitalization and mechanical ventilation show fibrosing DAD (1). Whether or not this frequency of fibrosis extrapolates to recovered severe patients remains to be established in future prospective studies. However, the development of lung fibrosis is a major concern in patients that have recovered from severe COVID-19 pneumonia. It has been reported that among survivors of severe COVID-19, 20% of non-mechanically ventilated and 72% of mechanically ventilated individuals had fibrotic-like radiographic abnormalities 4 months after hospitalization. The risk factors associated with its development were greater initial severity of illness, longer duration of mechanical ventilation and shorter blood leucocyte telomeres (2). Currently, despite the efforts of the community, including different clinical trials, there are no treatments for COVID-19 induced pulmonary fibrosis (3). Thus, lung transplantation is becoming a life-saving therapeutic option for patients with end-stage lung disease due to COVID-19 (4).

At present, the cellular components and molecular mechanisms of fibrosing DAD in COVID-19 patients are poorly understood (5–7). Although it is likely that the same cells and/or pathways described in other forms of lung fibrosis also participate in SARS-CoV-2 induced DAD. Recent studies have reported that fibrosis in both idiopathic pulmonary fibrosis (IPF) and COVID-19 can be driven, at least in part, by “pathological fibroblast” (pFB), characterized by the expression of a specific set of genes (8–10). Thus, pFB, together with other types of fibroblasts, seem to be the cellular effectors of lung fibrosis. They are driven under the stimuli of several pathways, such as the transforming growth factor beta (TGF-β), WNT, HIPPO or epithelial to mesenchymal transition (EMT) (11, 12). Accordingly, a few recent reports using single cell RNAseq technology have observed similarities in gene expression across cell lineages between end stage COVID-19 lungs and lungs of patients with pulmonary fibrosis (8, 10).

In this study, we describe the morphological, immunohistochemical and transcriptomic changes in the lungs of 33 COVID-19 patients, most with a prolonged clinical course, in order to evaluate the mechanisms of progression to fibrosis that would suggest possible new therapeutic strategies in patients with severe disease.

## Materials and methods

### Patients and tissue sample collection

This postmortem study included lung samples obtained from 33 COVID-19 patients. Autopsies were performed at the Hospital Universitario Ramón y Cajal (Madrid, Spain) between April 2020 and June 2021, as previously reported (13). The Research Ethics Committee approved the study (reference: Necropsias_Covid19; 355_20). Clinical, laboratory and radiological records were reviewed, and the main pathologies and treatments were recorded.

The autopsies represented about 3% of COVID-19 deaths during this period in our center. Most autopsies corresponded to patients with severe respiratory diseases and were requested by ICU staff. Consequently, the series does not represent the complete spectrum of causes of death attributable to COVID-19. All autopsies were consented by the patients’ relatives and carried out according to safety protocols, in a negative pressure autopsy room, using personal protection equipment, as previously reported (13). All autopsies were performed less than 24 h after patient’s death.

In the first 14 consecutive decedents, in-corpore representative sections were taken from the heart, lungs, liver, kidney, pancreas, and bone marrow. In the rest of the patients, due to improved technical training, the complete heart and lung block, left kidney, spleen and sections from the liver, pancreas and bone marrow were extracted. One autopsy was limited to the lungs.

For comparison, biopsy samples from 4 patients with typical lesions of usual interstitial pneumonia/idiopathic pulmonary fibrosis (UIP/IPF) were studied (Supplementary Table 1). UIP lesions met the criteria recently proposed by the 2018 ATS/ERS/JRS/ALAT Guidelines (14), including dense fibrosis with architectural distortion, predominant subpleural and/or paraseptal distribution of fibrosis, patchy involvement of lung parenchyma by fibrosis, presence of fibroblast foci and absence of features to suggest an alternate diagnosis.

In addition, 7 lung samples obtained from lung resections for different conditions other than UIP/IPF were used as normal controls. We selected areas far away from the pleura, or the specific pathological lesion in each case, that had a completely normal morphological appearance. We did not select autopsy samples as normal controls because in our institution the time of lung fixation of autopsy specimens is about 1 week, whereas lung fixation of COVID-19 specimen was between 24–72 h. Long fixation times have a negative impact on transcriptomic analysis.

### Lung lesions semiquantitative evaluation

Samples from the five pulmonary lobes were taken in all patients. All histological evaluations were blinded to clinical data. The histopathological classification of the DAD lesions was performed according to Li et al. (1) as previously reported (15).

The semiquantitative evaluation of lung lesions was performed on hematoxylin and eosin (H&E) and Masson’s trichrome stained sections. Ten to twenty sections were evaluated in each patient (at least 2 H&E sections from each lobe). The percentage of the section area occupied by each type of lesion was recorded. For quantitation, lesions were classified as acute/exudative, organizing/proliferating, and fibrotic. Acute/exudative lesions included massive epithelial desquamation, hyaline membranes, and intra-alveolar fibrin deposition; organizing/proliferating lesions included septal, and/or alveolar proliferation; fibrotic lesions included septal, and/or alveolar fibrosis, characterized by the presence of dense interstitial eosinophilic fibrous tissue green-stained by Massonn’s trichrome. Other changes, such as alveolar edema, capillary congestion, alveolar hemorrhage, squamous metaplasia, pleural fibrosis, etc. were recorded in each patient, but not quantitated for this analysis.

### Immunohistochemistry

The EnVision FLEX/HRP system was used for immunohistochemistry analysis (Agilent, Santa Clara, CA). For dual immunostainings, EnVision FLEX DAB + Chromogen (Agilent) was used to obtain the brown color and EnVision FLEX HRP Magenta Chromogen was used to obtain the magenta color. Antibodies, clones, dilutions, and providers are presented in Supplementary Table 2.

### RNA extraction

RNA was extracted from 10 tissue sections of 5 μm obtained from representative paraffin blocks. The Recover All Total Nucleic Acid Isolation Kit was used for formalin-fixed paraffin-embedded (FFPE) tissue (Invitrogen) following the manufacturer’s instructions. Quantification of RNA was measured fluorometrically with the Qubit RNA high-sensitivity assay kit (Invitrogen). RNA quality was assessed using RNA Screen Tapes on a 2200 TapeStation system (Agilent, Santa Clara, CA, USA).

### Gene expression analysis

The Nanostring nCounter gene expression platform was used to analyze the expression of 770 human mRNAs associated with fibrotic diseases included in the Fibrosis Panel (16). Fluorescently color-coded reporter probes and biotin-labeled capture probes were hybridized to the mRNA on a thermal cycler overnight and automatically processed and loaded to the nanoString sample cartridge in the nCounter Prep Station, in accordance with the manufacturer’s protocol.

The identification of differentially expressed genes was performed with normalized data using the nSolver analysis software (nanoString technologies Inc.). Specifically, the advanced analysis tool of this software allowed us to obtain the hierarchical grouping, the scatter diagrams (volcano plots) and statistical classification of the differentially expressed genes, along with FDR corrected *p*-values.

qRT-PCR was used to validate the expression of selected genes included in the Fibrosis Panel: *COL3A1*, *LOX* (collagen biosynthesis), *SPP1* [extracellular matrix(ECM) degradation], *TGF*β*1*, *SMAD3*, *TGFBR2* (TGF-β), *SNAI2* (Slug, EMT), *CXCL12* (chemokine signaling), *AMOLT1*, *MOB1B, TPJ1* (Hippo pathway). In addition, qRT-PCR was used to evaluate the expression of *CTHRC1*, *SPARC* and *YAP1*, not included in the Fibrosis Panel (Supplementary Table 4). The expression of these genes was also analyzed by IHC in 20 COVID-19 lung samples, normal controls and IPF samples.

Retrotranscription was performed with the High-Capacity cDNA Reverse Transcription Kit (Thermo Fisher Scientific), following the manufacturer’s instructions. The iTaq Universal SYBR Green Supermix Kit (BIORAD) was used following the manufacturer’s instructions. Data analysis was performed by quantifying the expression levels of the indicated genes, using a relative quantification by ΔΔCt method. The reference gene used was *GAPDH*.

### Sars-Cov-2 detection

Sars-Cov2 detection was performed as previously reported (17). Briefly, it was performed in post-mortem FFPE tissue from the lungs (all lobes), heart, liver and kidney in all patients. Genomic Sars-Cov-2 RNA (gRNA), and subgenomic viral RNA (sgRNA) were detected by RT-PCR.

### Statistical analysis

Statistical analyses were performed with R3.6.2 (18). Means comparisons were tested using the Student’s *t*-test and correlations using the Spearman’s coefficient. A *p* < 0.05 was considered statistically significant.

## Results

Some clinical, pathological and virological data from 26 patients have been previously reported (17).

### Clinical and laboratory data

This series included 26 males and 7 females, ranging in age from 52 to 91 years. The median disease duration was 36 days (IQR 17). A summary of the main clinical and laboratory data is presented in Table 1.

**TABLE 1:** Main clinical and laboratory data of the patients included.

Demographics and clinical characteristics			Total number of observations
Age, years	Median (IQR)	69 (13)	33
	Min, max	52, 91	
Gender, *n* (%)	Male	26 (78.8)	33
Weight, kg	Median (IQR)	76.5 (14)	28
	Min, max	53, 109	
DM, *n* (%)		4 (12.1)	33
Hypertension, *n* (%)		13 (39.4)	33
Patients admitted to ICU, *n* (%)		29 (87.9)	33
Total days	Median (IQR)	36 (17)	33
	Min, max	9, 108	
Hospitalization days	Median (IQR)	28 (16)	33
	Min, max	3, 102	
ICU days	Median (IQR)	23 (13)	29
	Min, max	12, 95	
Mechanical ventilation, *n* (%)		28 (84.5)	33
Corticosteroids use, *n* (%)		31 (93.9)	33
Immunomodulatory therapy*, *n* (%)		28 (84.8)	33
**Lung pathological findings**			
Patients with predominant pattern, *n* (%)			
	Normal lung	2 (6.1)	33
	Exudative DAD	6 (18.2)	33
	Proliferative/ Organizing DAD	21 (63.6)	33
	Fibrotic DAD	4 (12.1)	33
Vascular thrombi, *n* (%)		22 (66.7)	33
Endothelialitis, *n* (%)		15 (45.5)	33
**Lung infections**			
Acute bronchopneumonia, *n* (%)		8 (24.2)	33
Aspergillosis, *n* (%)		3 (9.1)	33
Cytomegalovirus, *n* (%)		2 (6.1)	33
**Main pathological findings in other organs**			
**Heart**			
No lesions, *n* (%)		13 (40.6)	32
Coronary artery atherosclerosis, *n* (%)		11 (34.4)	32
Left ventricle hypertrophy, *n* (%)		4 (12.5)	32
Chronic epicardial inflammation, *n* (%)		4 (12.5)	32
Chronic ischemic cardiopathy, *n* (%)		2 (6.3)	32
Myocarditis, *n* (%)		1 (3.1)	32
Senile amyloidosis, *n* (%)		1 (3.1)	32
**Liver**			
No lesions, *n* (%)		11 (34.4)	32
Steatosis, *n* (%)		11 (34.4)	32
Centrilobular necrosis, *n* (%)		4 (12.5)	32
Cirrhosis, *n* (%)		1 (3.1)	32
**Kidney**			
No lesions, *n* (%)		5 (15.6)	32
Ischemic necrosis, *n* (%)		10 (31.3)	32
Acute tubular necrosis, *n* (%)		10 (31.3)	32
Glomeruloesclerosis, *n* (%)		4 (12.5)	32
**Bone marrow**			
No lesions, *n* (%)		1 (3.1)	32
Hyperplasia, *n* (%)		30 (93.8)	32
Hemophagocytosis, *n* (%)		25 (78.1)	32

*Including tocilizumab and/or interferon β1a.

### Lung pathology

**FIGURE 1:**
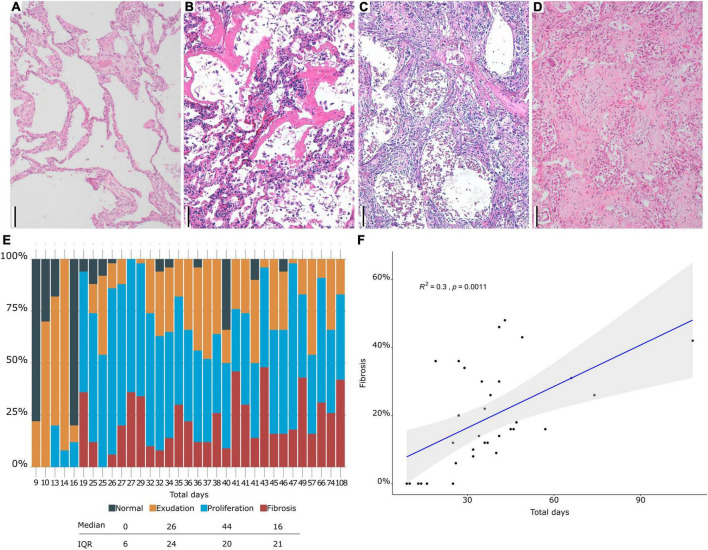
**(A–D)**. Histological evaluation. **(A)** Normal lung. **(B)** Hyaline membranes in exudative diffuse alveolar damage (DAD). **(C)** Proliferative DAD. **(D)** Fibrosing DAD. **(E)** Proportion of different DAD patterns (y axis) in each patient (x axis). **(F)** Correlation between fibrosis and time of evolution. Scale bar: 100 μm.

**FIGURE 2:**
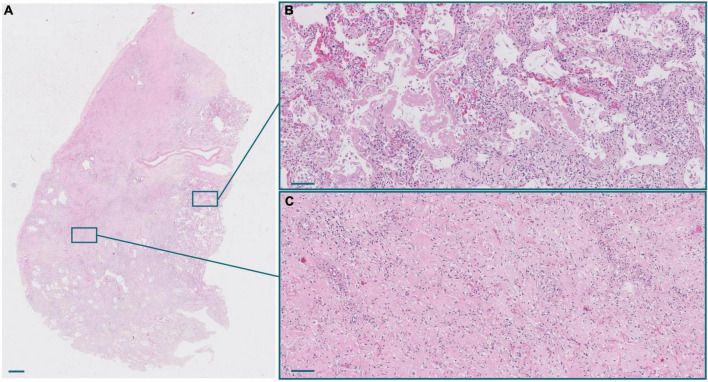
**(A)** Panoramic view of a lung section from a patient with a long clinical course. Scale bar: 1 mm. **(B)** Inset of an exudative area, with hyaline membranes. Scale bar: 100 μm. **(C)** Inset of a fibrotic area. Scale bar: 100 μm.

Macroscopic examination of the lungs showed increased consistency and poor aeration with consolidated areas in all cases, which varied in extension from patient to patient. The lungs showed a spectrum of histological lesions, both acute and chronic, consisting off desquamated pneumocytes, capillary congestion, pulmonary edema, hyaline membranes, intralveolar fibrin deposition, septal fibroblastic proliferation, intralveolar fibroblastic proliferation, septal fibrosis, intralveolar fibrosis, and squamous cell and mucinous metaplasia of the alveolar epithelium. Multiple combinations of these lesions were observed in most patients, making it difficult to assign a specific diagnosis to each patient. DAD in different evolution stages was present in all patients (Figures 1, 2). Thus, patients with a prolonged clinical course showed overlapping exudative, proliferative, and fibrotic lesions (Figure 2). The exudative pattern predominated in 6 patients (18.2%), the proliferative pattern in 21 (66.7%) and the fibrotic pattern in 4 patients (12.1%). Mean fibrosis was 16% (0–44%). Although there was a direct correlation between fibrosis and illness duration (Figure 1), the percentage of fibrosis varied among patients with a similar duration of illness (see Supplementary Table 3).

The most common inflammatory cells were CD68-positive alveolar macrophages. The proportion of these cells varied among patients, but were abundant, even in patients with a longer illness duration. In addition, all patients showed variable number of CD8-positive lymphocytes that were more abundant than CD4-positive lymphocytes.

Histological changes concordant with previous lung damage were observed, such as increased pleural fibrosis with muscularization and honeycomb changes in 3 patients (patient 12, 16, and 20), and silicosis nodules in 1 case (patient 16). None of these patients had a previous clinical history of chronic lung disease.

### Sars-Cov-2 RNA detection

**FIGURE 3:**
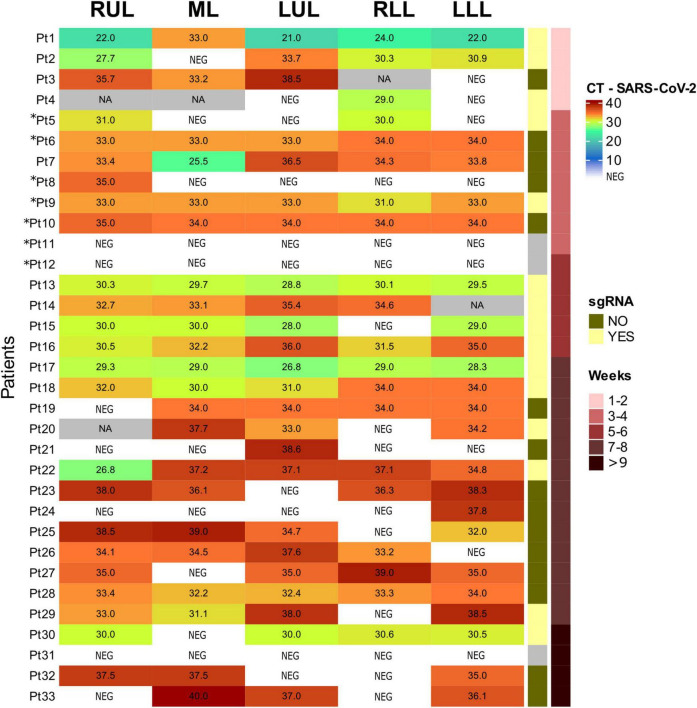
Sars-Corv-2 gRNA and sgRNA in lung samples. RUL, right upper lobe; ML, medium lobe; LUL, left upper lobe; RLL, **right lower** lobe; LLL, **left lower** lobe; CT, cycle threshold; NEG, negative; NA, non-available; sgRNA, subgenomic RNA. Patients not included in (17) are 5, 6, 8, 9, 10, 11, and 12 (with asterisks).

A detailed description of Sars-Cov2 results in lung samples is presented in Figure 3. In summary, Sars-Cov2 gRNA and sgRNA were detected in the lung of 27 and 15 patients, respectively. Sars-Cov2 gRNA was even detected in samples from the patients with a longer disease evolution (104 days). Regarding other organs, viral gRNA was detected only in the heart of Pat. 1. gRNA Cts-values in positive samples ranged from 21 to 40. sgRNA was detected in samples from 15 patients (50%).

### Fibrosis-associated gene expression

Transcriptomic analysis using the NanoString platform was performed in 12 COVID-19 samples with RNA of sufficient quality. Samples originated from patients in their third to seventh week of disease evolution. In each case, we selected for Nanostring analysis those histological sections in which proliferative DAD predominated, assuming that it was the disease phase in which fibrogenesis was more active. This phase of DAD was also selected because previous studies on gene expression analysis have mainly focused on patients with a shorter evolution in which exudative DAD predominated, and this type of sample were underrepresented in our series. In addition, 7 normal lung controls were analyzed.

**FIGURE 4:**
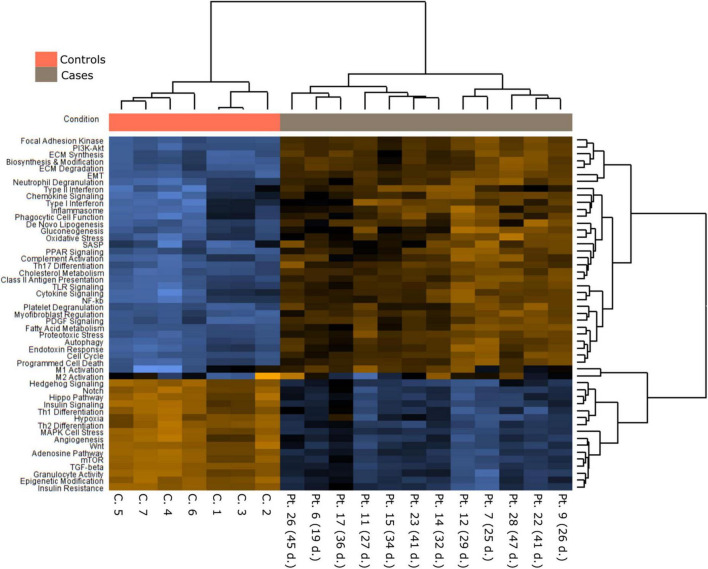
A Pathways differently expressed in normal and COVID-19 samples.

**FIGURE 5:**
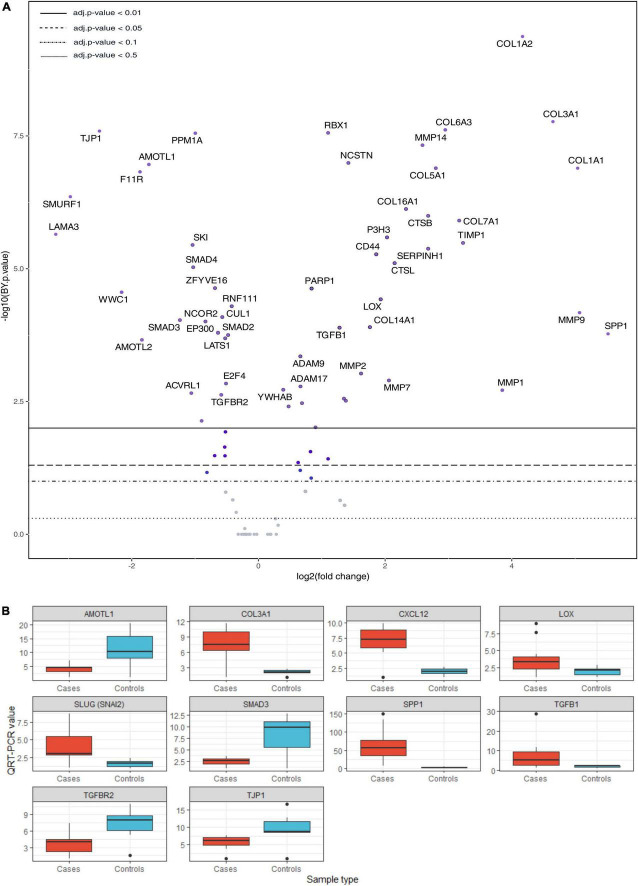
**(A)** Volcano plot showing differentially expressed genes of collagen biosynthesis, extracellular matrix biosynthesis and degradation, TGF-β and Hippo pathways. **(B)** Validation by qRT-PCR. *T*-test: AMOTL1 *p* = 0.031, COL3A1 *p* = 0.00028, CXCL12 *p* = 3.9 e-05, LOX *p* = 0.027, SLUG (SNAI2) *p* = 0.0052, SMAD3 *p* = 0.014, SPP1 *p* = 0.00083, TGFB1 *p* = 0.043, TGFBR2 *p* = 0.029, TJP1 *p* = 0.072. QRT-PCR units: ΔΔCt.

**FIGURE 6:**
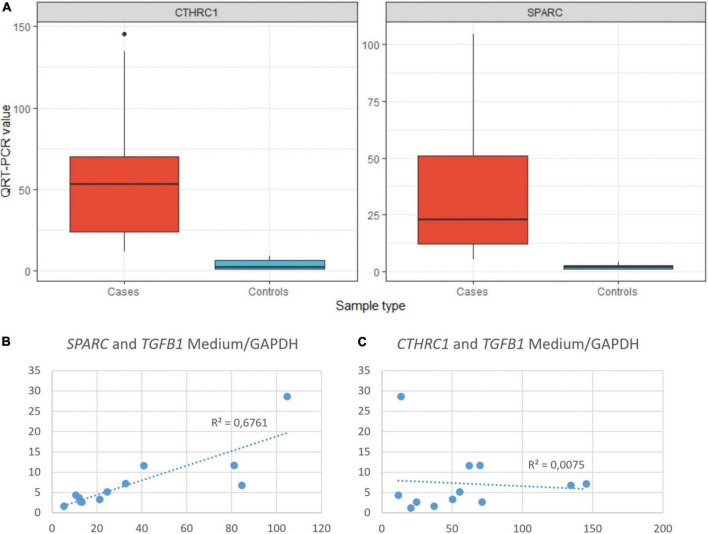
**(A)** Statistical differences in the expression of CTHRC1 and SPARC between COVID-19 (cases) and normal (controls) samples. *T*-test: CTHRC1 *p* = 0.0012, SPARC *p* = 0.0054. QRT-PCR units: ΔΔCt. **(B)** Correlation between the expression of SPARC and TGFβ1 in COVID-19 samples. **(C)** Correlation between the expression of CTHRC1 and TGFβ1 in COVID-19 samples.

Figures 4–6, Supplementary Figure 1, and Supplementary Table 5 present the main pathways and genes differentially expressed between cases and healthy lung controls. The gene expression study confirmed the histological observation of increased fibrogenesis in COVID-19 patients, as genes related with collagen biosynthesis and ECM biosynthesis and degradation, such as *COL1A2*, *COL3A1*, *COL6A3*, *COL1A1*, *COL5A1*, *SPP1*, *MMP14*, *NCSTN*, and *LOX* were overexpressed in COVID-19 patients.

**FIGURE 7:**
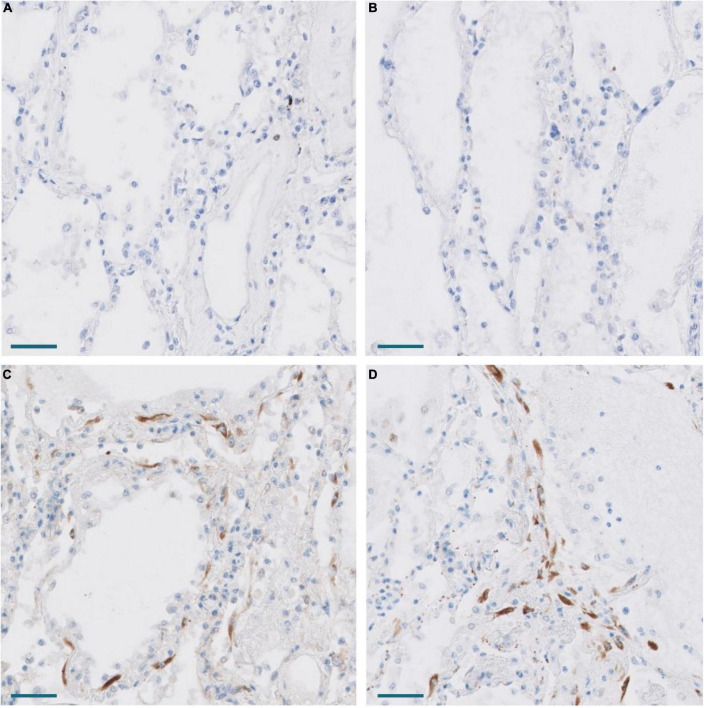
Expression of CTHRC1 and SPARC in the lung of Pt. 3 with 13 days evolution. **(A,B)** No expression of CTHRC1 and SPARC in normal lung areas. Scale bar: 100 μm. **(C,D)** CTHRC1 **(C)** and SPARC **(D)** expression in interstitial fibroblasts in areas with early exudative diffuse alveolar damage. Scale bar: 100 μm.

**FIGURE 8:**
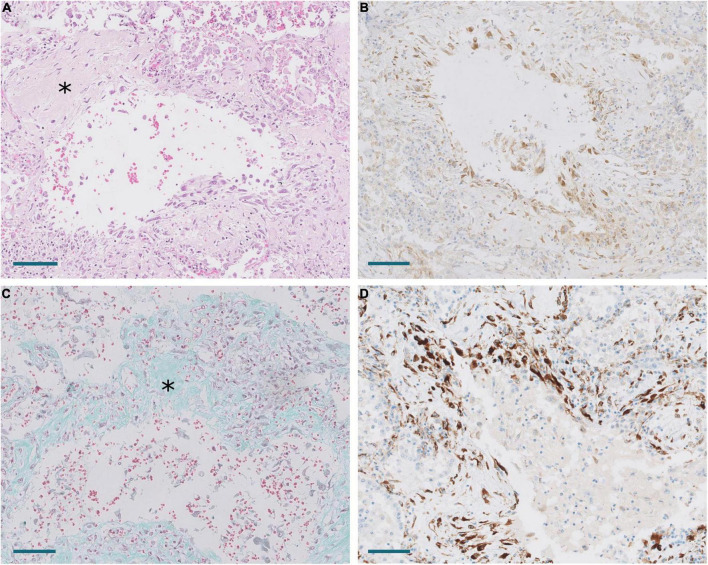
Areas of periductal proliferation/fibrosis in Pt.18 with 43 days of disease evolution. **(A)** Hematoxylin and eosin. Note deposition of collagen (*). Scale bar: 100 μm. **(B)** Expression of CTHRC1. Scale bar: 100 μm. **(C)** Masson trichrome stain. Note collagen deposition in green (*). Scale bar: 100 μm. **(D)** SPARC expression. Scale bar: 100 μm.

**FIGURE 9:**
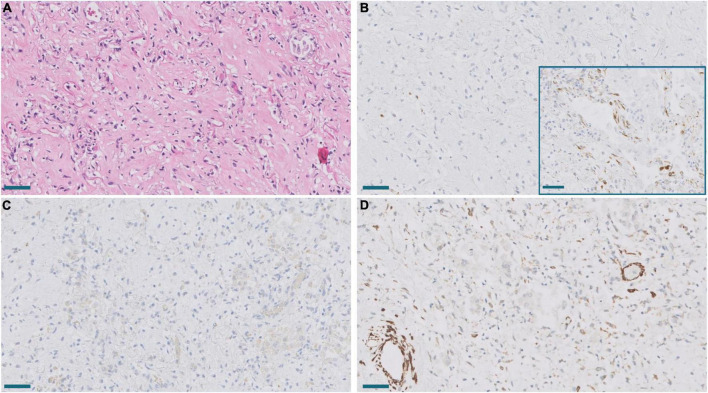
**(A)** Established area of mature fibrosis (HE). Scale bar: 100 μm. **(B)** Lack of expression of SPARC in mature fibrosis in contrast to moderate SPARC expression in an active proliferative area (inset). Scale bar: 100 μm. **(C,D)**. Lack of CTHRC1 expression **(C)** and diminished α-muscle actin expression **(D)** in the same fibrotic area. Scale bar: 100 μm.

As previously mentioned, some studies have reported that fibrosis in both IPF and COVID-19 can be, at least in part, driven by pFB (also called activated fibroblasts in some articles) characterized by the expression of a set of genes, such as *CTHRC1*, *COL3A1*, and *COL1A*. Among these genes, *CTHRC1* has been proposed as one of the most specific (7). Since our panel did not include *CTHRC1*, its expression was analyzed by both RT-PCR and IHC. There was a statistically significant increase of *CTHRC1* expression in COVID-19 samples when compared with control samples (Figure 6). In fact, the level of expression in normal appearing control lungs was negligible. By IHC, cells expressing CTHRC1 were not present in control lungs. In contrast, positive cells were first observed in areas of acute DAD (Figure 7). CTHRC1-positive cells increased in areas of fibroblastic proliferation and organization (Figure 8) and were scarce or absent in areas of mature fibrosis (hypocellular areas mainly composed by dense ECM) (Figure 9).

Melms et al. (10) observed in their single cell RNAseq analysis of COVID-19 lung samples that *SPARC* was the second most upregulated gene in pFB. This prompted us to study *SPARC* in our series by RT-PCR and immunohistochemistry, since this gene was not included in the NanoString panel. We observed a statistically significant increase of *SPARC* expression in COVID-19 samples compared with control samples. Like *CTHRC1*, the level of expression in control lungs was very low. By immunohistochemistry, we also observed that SPARC-positive fibroblasts were absent in normal lung, appeared in exudative DAD, peaked in areas of proliferative DAD and decreased in fibrotic DAD (Figures 7, 8). Areas of mature fibrosis were devoid of SPARC-positive cells (Figure 9).

**FIGURE 10:**
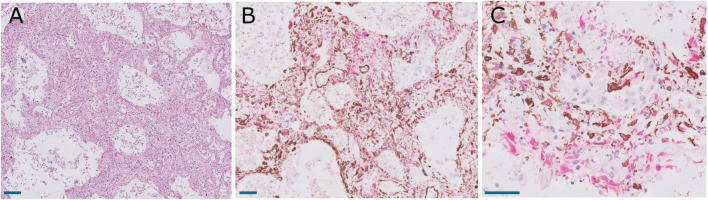
Lung lesions in Pt. 25 with 41 days of evolution. **(A)** Hematoxylin and eosin showing interstitial enlargement. Scale bar: 100 μm. **(B,C)**. Dual immunostaining with SPARC (magenta) and α-muscle actin showing a heterogeneous population of fibroblast expressing different levels of both proteins. Scale bar: 100 μm.

Immunostaining with α-muscle actin demonstrated that the population of positive cells were not identical to CTHRC1- and SPARC-positive cells. Thus, dual immunostaining with SPARC and α-actin in proliferative areas demonstrated a mixed population of cells expressing one or both markers at different levels (Figure 10). Fibroblast in areas of mature fibrosis showed no or very low α-actin expression (Figure 9).

An intriguing result in our study was the observation that the TGFβ pathway, a well-known fibrogenic pathway, was downregulated in our series of COVID-19 patients. However, both *TGF*β*1* and its downstream target SPARC (19) were overexpressed and showed an statistically significant direct correlation (not observed between *TGF*β*1* and *CTHRC1*) (Figure 6). Since genes such as *SMAD2*, *SMAD3*, and *SAMAD4* were hypoexpressed, our results suggested non-canonical activation of the TGFβ pathway. Interestingly, the PI3K-AKT signaling pathway, a common target of the non-canonical (non-Smad2/3) TGFβ pathway (20), was activated in our series (Figures 4, 5 and Supplementary Figure 1).

### COVID-19 fibrosis vs. usual interstitial pneumonia/idiopathic pulmonary fibrosis

**FIGURE 11:**
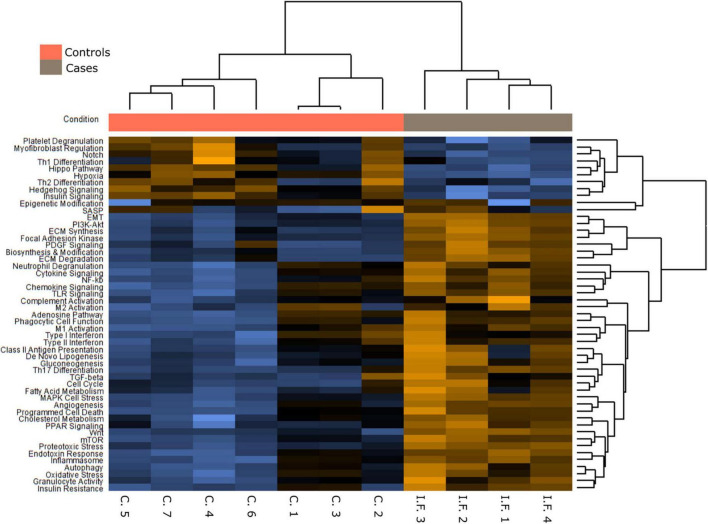
Pathways differentially expressed in idiopathic pulmonary fibrosis (IPF) in comparison with normal lung.

**FIGURE 12:**
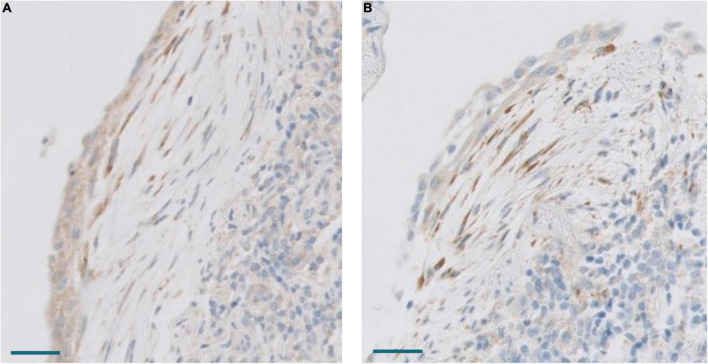
**(A)** Expression of CTHRC1 in a fibroblastic focus. Scale bar: 100 μm. **(B)** Expression of SPARC in the same fibroblastic focus. Scale bar: 100 μm.

In order to analyze possible similarities between proliferative DAD in COVID-19 patients and UIP/IPF, the transcriptomic profile of biopsies obtained in 4 UIP/IPF patients were analyzed. When compared to normal lung samples, UIP/IPF samples showed up- and down-regulation of similar pathways to those observed in COVID-19 patients. Thus, genes related with collagen biosynthesis and ECM biosynthesis and degradation were up-regulated in UIP/IPF samples (Figure 11). In fact, overexpression of some genes involved in these processes, such as *COL1A2* and *COL3A1*, were observed in both COVID-19 and UIP/IPF. In general, there was a higher expression in COVID-19 samples, indicating more fibrogenesis in COVID-19-associated DAD (Supplementary Table 6). Immunohistochemical analysis in our cohort demonstrated that CTHRC1 and SPARC expression was characteristic of pFBs in fibroblast foci (Figure 12).

### Epithelial to mesenchymal transition in COVID-19 patients’ fibrosis

**FIGURE 13:**
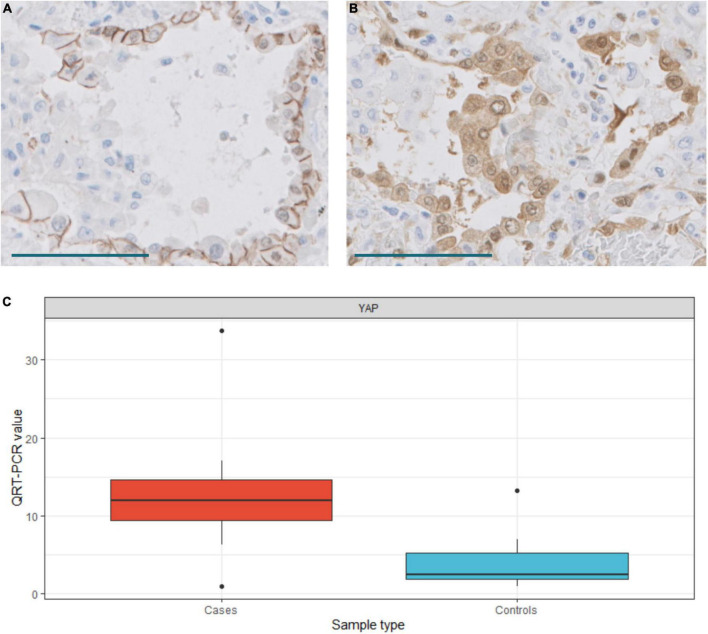
**(A)** Cadherin 11 expression in hyperplastic pneumocytes. Scale bar: 100 μm. **(B)** YAP expression in hyperplastic pneumocytes. Scale bar: 100 μm**. (C)** Differences in YAP expression between normal (control) and COVID-19 (case) samples. *T*-test, *p* = 0.0076. QRT-PCR units: ΔΔCt.

EMT has been proposed as a mechanism to recruit fibroblast in some fibrogenic lung conditions (11). True EMT is a complex process that implies that epithelial cells lose their epithelial characteristics and acquire a mesenchymal phenotype (21). Our gene expression analysis indicated that the EMT process was upregulated in the lungs of COVID-19 patients (Figures 4, 5). However, genes involved in EMT are usually expressed in fibroblasts and other mesenchymal cells, which were increased in number in COVID-19 lungs. To test if epithelial lung cells modified their epithelial phenotype to transform into fibroblasts, we analyzed cadherins and catenins by immunohistochemistry since they were not included in the Nanostring panel. The first step of EMT is cadherin switching, by which epithelial cells lose E-cadherin and acquire a mesenchymal cadherin (such as N-cadherin or cadherin 11), which is usually accompanied by the cytoplasmic relocation of catenin, such as p120-catenin. E-cadherin and cadherin 11 were expressed in normal bronchial, bronchiolar, and alveolar epithelial cells. In addition, they were expressed in hyperplastic epithelial alveolar cells in COVID-19 patients. Due to cell enlargement, the expression of cadherin 11 was more evident in these hyperplastic cells (Figure 13). β-catenin and p120 were expressed in epithelial cells, fibroblasts, and endothelial cells in healthy lungs. No evident changes were observed in the location of these molecules in epithelial cells of COVID-19 patients. Specifically, no nuclear expression of β-catenin was observed in any cell type and p120 was mainly expressed in the cell membrane without evident relocation into the cytoplasm (not shown). Moreover, Slug, a transcriptional repressor of E-Cadherin that was upregulated in our transcriptomic analysis (Figure 5), was expressed in some fibroblasts in COVID-19 patients but not in epithelial cells (not shown). These data argue against a role of EMT in the process of recruiting pFB in DAD in COVID-19 patients.

### Overexpression of the Hippo pathway

The Hippo pathway has been implicated in lung fibrosis (22), but it’s possible role in COVID-19 lung pathology has not been analyzed. Our transcriptomic analysis indicated that the Hippo signaling pathway was downregulated in COVID-19 patients. Thus, several genes involved in the Hippo pathway were downregulated in our NanoString analysis and these results were confirmed by RT-PCR (Figures 4, 5). The inhibition of the Hippo pathway leads to the activation of its effector protein YAP, which has been implicated in both normal lung development and IPF. By RT-PCR, there was a statistically significant increase of *YAP* expression in COVID-19 lung samples compared with controls. By IHC, mild YAP expression was observed in alveolar epithelial cells and in the basal cells of bronchial epithelium of normal lung samples. Increased nuclear and cytoplasmic YAP expression was mainly observed in hyperplastic pneumocytes of COVID-19 samples (Figure 13). These data suggest that Hippo signaling is modulated in epithelial cells in COVID-19 patients.

## Discussion

This series included 33 COVID-19 patients with a prolonged illness (median 36 days), hospitalization (median 28 days) and mechanical ventilation (median 23 days). We demonstrated that, although proliferating DAD predominated, some degree of fibrosing DAD was present in 27 patients with more than 2 weeks of disease evolution, being the predominant pattern in 4 patients (12%). Few autopsy series have analyzed the evolution to fibrosis in COVID-19 patients. Li et al. (1) analyzed a cohort of 30 minimally invasive autopsies with a mean illness duration of 43 days and observed that fibrosis was the predominant pattern in 43% of the patients. However, this high incidence might be an overestimation since the tissue was obtained by ultrasound-guided core biopsy of selected areas. Regarding fibrosis extension, this reached a maximum of 48%. Similarly, Melms et al. (10) analyzed a series of 16 autopsies with a mean illness duration of 28 days and observed a maximum of 30% of fibrosis in some patients.

Although the presence of fibrosis correlated with the duration of illness, hospitalization and ICU stay, important differences were observed among patients with a similar evolution time. These differences were probably related to different factors, such as SARS-CoV-2 persistence, previous lung conditions, superimposed infections and the ventilatory management of each patient. In this sense, we must stress that exudative DAD was present in most patients, even in those with prolonged evolution. Thus, indicating the existence of persistent lung injury in most patients.

The detection of Sars-Cov2 RNA in most lung samples, but not in other organs in this series (17), suggests a role of viral persistence in the maintenance of lung injury in COVID-19 patients. It is reasonable to speculate that viral persistence could be partially due to a defective viral clearance by impaired host immunity in this cohort of aged patients, most of them with lymphopenia and subjected to immunosuppressive therapy such as corticoid.

In order to gain an insight into the mechanisms involved in the progression of lung lesions, we performed a transcriptomic and immunohistochemical analysis of lung samples. Concordant with our morphological observations, we observed overexpression of genes involved in the fibrogenic processes such as collagen biosynthesis and ECM remodeling in COVID-19 samples. Lung fibroblasts represent a heterogenous population of cells whose diversity is being elucidated by sc-RNA seq analysis. Tsukui et al. (9) identified a subpopulation of pFB characterized by the expression of *COL1A1* and *COLA31* in IPF samples (two of the genes highly overexpressed in our NanoString analysis in COVID-19 samples) and other ECM genes such as *SPP1* (also overexpressed in our COVID-19 samples) or *TNC* (not included in our panel). Among ECM genes, *CTHRC1* was the most specific for pFB. An enrichment of *COLA31* + */CTHRC1* + pFB has also been observed in some COVID-19 samples (8, 10, 23). However, the temporo-spatial expression of CTHRC1 + cells has not been previously described in these patients. We observed a progressive increase of CTHRC1 + cells from normal lungs, in which they were absent, to proliferative DAD. Their increased frequency during this period suggested that CTHRC1 + cells were pFBs promoting rapidly evolving lung fibrosis in individuals with COVID-19. CTHRC1 + cells were initially located in alveolar septa and then distributed in areas of septal and periductal fibrosis. As previously suggested in IPF, CTHRC1 + cells seemed to respond to alveolar injury by migrating into injured areas where they participated in tissue fibrosis by producing excess quantities of ECM (9).

Our study also suggested an important role of the fibrogenic factor SPARC in COVID-associated lung fibrosis. SPARC is a matricellular component of ECM. It has been demonstrated that lung fibroblasts isolated from IPF patients constitutively express more SPARC than those derived from subjects without IPF (24). Moreover, SPARC-null mice display a diminished degree of pulmonary fibrosis compared with control mice after exposure to bleomycin (19). In addition, it has been demonstrated that SPARC secreted by IPF fibroblasts acts as a paracrine signal promoting persistent alveolar epithelial activation, thus, preventing normal epithelial repair responses and restoration of tissue homeostasis (24). Whereas these previous observations clearly demonstrated that SPARC is involved in the development of IPF, its possible role in other pulmonary lesions, including COVID-19 DAD, was not previously evaluated in deep. Although the expression of SPARC was consistently increased in some sc-RNA-Seq datasets of COVID-19 patients (10), no previous studies have analyzed its expression during the different phases of DAD nor its tissue distribution. Our dual immunostaining study with an anti-α-actin antibody indicated that some, but not all, SPARC-positive fibroblast also expressed different amounts of α-muscle actin, suggesting different functional states among the population of pathological (or activated) fibroblast. Our study also demonstrated that areas of mature fibrosis were devoid of cells expressing CTHRC1, SPARC or α-actin, suggesting that loss a “pathological” or “activated” fibroblast phenotype was associated with fibrosis maturation, as previously reported during heart infarct scar maturation (25).

We compared the transcriptomic profile of proliferative DAD in COVID-19 with that observed in a group of UIP/IPF lesions and found up- and down-regulation of similar pathways in both conditions. Thus, genes related with collagen biosynthesis and ECM biosynthesis and degradation were up-regulated in both COVID-19 and UIP/IPF. In addition, a population of CHTR1 + and SPARC + fibroblasts were present in both active proliferative areas of DAD and in fibroblastic foci of UIP/IPF. These results indicate that, despite differences in etiology, time of evolution and morphology of lesions, the fibrotic process in both entities is associated with a similar transcriptomic program and with the activation of a similar population of fibroblasts. Accordingly, some studies have suggested the use in COVID-19 patients of anti-fibrotic drugs currently approved for the treatment of IPF (26).

EMT has been implicated in lung pathologies as a mechanism to promote fibrosis (11, 27). The study of cadherins and catenins in this series seems to exclude EMT as an mechanism of epithelial transdifferentiaton to fibroblasts in COVID-19 DAD, as also suggested in IPF (11, 28). Cadherin 11 is a mesenchymal cadherin whose expression in epithelial cell is usually related with EMT. An interesting observation in our study was the constitutively co-expression of cadherin 11 with E-cadherin in normal epithelial lung cells, which has not been previously reported. Accordingly, the expression of cadherin 11 in hyperplastic COVID-19 alveolar epithelial cells does not represent the cadherin switching process that initiates EMT. Both E-cadherin and cadherin 11, together with β-catenin and p120 showed a normal expression pattern in alveolar epithelial cells and other epithelial cells in COVID-19 patients. Whereas these data seemed to exclude the acquisition of a mesenchymal (fibroblastic) phenotype by epithelial cells, this does not preclude a paracrine role of epithelial cells in the promotion of fibrosis through the production of growth factors after the activation of EMT program (11, 27). The immunohistochemical analysis of β-catenin supported our transcriptomic results suggesting a minor role of the canonical WNT pathway in fibrogenesis, since no nuclear β-catenin expression was observed in any cell population. Our data are in accordance with those reported by Chilosi et al. (29), where nuclear β-catenin was not observed in pulmonary diseases such as DAD, organizing pneumonia, non-specific interstitial pneumonia and desquamative interstitial pneumonia.

In our transcriptomic study, we observed down-regulation of the Hippo pathway. To gain insights into the main cellular component implicated in the modulation of the Hippo pathway, we performed an IHC analysis of YAP expression, one of the main effectors of this pathway. We observed overexpression of YAP mainly in hyperplastic epithelial alveolar cells, suggesting a role of the Hippo pathway in mediating the epithelial lung response after SARS-COV2 infection. Our results confirm and expand previous observations indicating that the Hippo signaling modulates alveolar regeneration after acute lung injury (30). During embryogenesis, YAP is expressed in progenitor basal cells and controls airway epithelial differentiation (31, 32). When these progenitor cells are subjected to acute injury, YAP localization shifts from the cytoplasm to the nucleus and proper differentiation does not occur, resulting in epithelial hyperplasia and stratification (33). The Hippo signaling pathway is also modulated in epithelial cells in IPF, where nuclear YAP is expressed in epithelial cells (22). Abnormal regulation of the Hippo pathway has been observed during infection with a variety of viruses such as HBV, HCV, MCV, ZIKV, EBV, KSHV, HPV, and MuPyV (34). Recently, it has been reported that several proteins involved in the Hippo pathway, including YAP, can be targeted by the SARS-CoV-2 protease 3CL^pro^ (35). Although the exact role of SARS-COV2 in the modulation of the Hippo pathway remains to be established, our study suggests that deregulation of the Hippo pathway may contribute to the dramatically altered cell morphology in SARS-CoV-2–infected epithelial cells in the lungs of COVID-19 patients.

In summary, our study shows that progression to fibrosis in severe COVID-19 was associated with overexpression of fibrogenic pathways and increased in CTHRC1- and SPARC-positive pFB. Whereas the Hippo pathway seemed to be implicated in the response to epithelial cell damage, EMT was not a major process involved in COVID-19 mediated lung fibrosis. A possible role of viral persistence in the maintenance of lung damage is therefore suggested.

## Data availability statement

The datasets presented in this study can be found in online repositories. The names of the repository/repositories and accession number(s) can be found below: https://www.ncbi.nlm.nih.gov/geo/, GSE206788.

## Ethics statement

The studies involving human participants were reviewed and approved by the Research Ethics Committee, Ramón y Cajal University Hospital, approved the study (reference: Necropsias_Covid19; 355_20). Written informed consent for participation was not required for this study in accordance with the national legislation and the institutional requirements.

## Author contributions

JP and BP-M: study design. TB, JR-B, RP, and DP: clinical data collection. BP-M, JP, IC-B, AB, MG-C, IG-G, and MG-C: pathological evaluation. TC-C, DP, and YR: transcriptomic analysis. MR and EC: tissue processing. JG: viral analysis. IC-B, DP, and TC-C: statistical analysis. JP, BP-M, and IC-B: drafting the manuscript. All authors: discussion and final approval of manuscript.

## Supplementary material

Supplementary Figure 1 **(A)** Volcano plot. Genes differentially expressed between COVID-19 and normal samples. **(B)** Bar graphs of some of differentially expressed pathways between COVID-19 and controls: collagen biosynthesis and modification, extracellular matrix (ECM) degradation, epithelial to mesenchymal transition (EMT), PI3K-Akt, Hippo pathway and TGF-β.

Supplementary Table 1 Clinicopathological characteristics of the controls used. Four controls were taken from IPF biopsies and 7 control from normal lung present in segmentectomies due to pneumothorax.

Supplementary Table 2 Antibodies, clones, dilutions, and providers.

Supplementary Table 3 Detailed information of the cases in the study.

Supplementary Table 4 Primers design for validation.

Supplementary Table 5 Results of Nanostring analysis, comparing COVID-19 samples and controls.

Supplementary Table 6 Common genes differentially expressed in COVID-19 and IPF/UIP samples when compared with normal samples.
